# The Marvelous Gum

**Published:** 1875-10

**Authors:** 


### THE MARVELLOUS GUM.

Daniel Webster, in his great argument in behalf of Goodyear, the inventor of the process of vulcanizing India-rubber, said that "to discover a method by which a soft, yielding, flexible, elastic gum, could in an hour be converted into a metal, was nothing less than an inspiration." He alluded to the process by which what is known as "hard-rubber," is created. But it is really surprising to notice to how many uses India-rubber, either fully or partially vulcanized, is subjected. We have spoken of the India-rubber Life-Preserving Suits, and of the India-rubber Exercising Tubes, and now we are glad to call attention to still another industry, which would be utterly impossible without this wonderful gum. We refer to the India-rubber types and stamps exhibited at the Fair, by Messrs. G. K. Cooke & Co., of No. 92 Chambers Street, New York. When we speak of these types, we are talking about something which ought to interest everybody, because there is not a man nor a woman, not a boy nor a girl, in the country, who would not enjoy the possession of a stereotype plate containing his or her name and address in India-rubber. In a hundred ways, such a plate and its inking-apparatus would be found useful. In writing a letter, the name and address can be imprinted upon the upper left-hand corner of the envelope, and this will secure the ultimate return of the letter if it be not delivered. The fly-leaf of a book may be stamped in like manner, and there will be no valid excuse for the borrower to withhold it from its owner. The linen may be legibly and indelibly marked, and comparative immunity against loss secured. The address, without the name, may be fixed to a stamp; and this may be used at the head of a letter, and save much writing. The names of the months may be employed in like manner, as well as the combination of figures denoting the year.

When we come to business uses, there are more ways in which these stamps can be employed than we could think of in a month. Every man who does business of any kind will find it greatly to his advantage to have several of these rubber stamps. He can mark packages and boxes without cost, and thus advertise his business constantly and inexpensively. He can produce his own business cards at the rate of several hundred impressions an hour, and can thus be largely independent of the printer's art. In short, there are a multitude of uses to which these types can be applied with satisfaction and profit.

One excellence of these stamps is that they do not fail to make a good impression. Their very elasticity secures this, regardless of the irregularity of the surface of the paper printed on. They are durable, not being perceptibly worn by 50,000 impressions.

In fact, we have been so well pleased with these stamps of Mr. Cooke's, since our careful examination of them, that we have thought very seriously of arranging with him to allow us to offer them as premiums for lists of subscribers. We know of nothing more attractive. A complete outfit of ink and pad and box, with a name occupying one line, ready for use, is sent by mail for 75 cents; and with name and address (two lines) for $1. Any kind of type will be used as desired. If our readers will select from any source at hand a neat letter, and enclose it with the money to the office of Mr. Cooke, they will shortly receive by mail what they order, and we are confident that they will be amply satisfied.

